# Novel end-to-side one-layer continuous pancreaticojejunostomy vs. end-to-end invaginated pancreaticojejunostomy in pancreatoduodenectomy: A single-center retrospective study

**DOI:** 10.3389/fsurg.2022.980056

**Published:** 2023-01-06

**Authors:** Dong Luo, Yixiong Li, Liandong Ji, Xuejun Gong

**Affiliations:** ^1^ Department of Hepatopancreatobiliary Surgery II, Third Xiangya Hospital, Central South University, Changsha, China; ^2^ Department of General Surgery (Pancreatic Surgery), Xiangya Hospital, Central South University, Changsha, China

**Keywords:** pancreaticoduodenectomy, pancreatic fistula, end-to-side pancreaticojejunostomy, one-layer continuous pancreaticojejunostomy, pancreatic anastomosis

## Abstract

**Background and Objective:**

Postoperative pancreatic fistula (POPF) is the most common critical complication after pancreatoduodenectomy (PD) and is the primary reason for increased mortality and morbidity after PD. We aim to investigate the clinical significance of a novel approach, i.e., end-to-side one-layer continuous pancreaticojejunostomy, for patients with PD.

**Methods:**

The clinical data of 65 patients who underwent pancreatoduodenectomy at the Xiangya Hospital, Central South University, from September 2020 to December 2021 were retrospectively analyzed.

**Results:**

Forty patients underwent end-to-end invaginated pancreaticojejunostomy, and 25 underwent the novel end-to-side one-layer continuous pancreaticojejunostomy. No significant differences were observed in pancreatic fistula, intraperitoneal infection, intraperitoneal bleeding, reoperation, postoperative hospital stay, or perioperative death between the two groups. However, the novel end-to-side one-layer continuous pancreaticojejunostomy group had significantly shorter operation duration (32.6 ± 5.1 min vs. 8.3 ± 2.2 min, *p *< 0.001). The incidence of pancreatic fistula in the novel pancreaticojejunostomy group was 12%, including two cases of grade A POPF and only one case of grade B POPF. No cases of grade C POPF occurred. No deaths were observed during the perioperative period.

**Conclusions:**

The novel anastomosis method leads to a shorter operation duration than the traditional anastomosis method and does not increase postoperative complications. In conclusion, it is a simplified and feasible method for pancreatic anastomosis.

## Introduction

Pancreaticoduodenectomy (PD) is a widely performed but challenging operation that involves multiple procedures to resect tumors in the periampullary region (pancreatic head and surrounding areas) (1, 2). The PD procedure has been a challenging operation since it was first performed in 1898 (3) and is characterized by high rates of perioperative mortality, morbidity, and postoperative complications (4, 5). In recent decades, efforts to optimize perioperative management, improve surgical techniques, and centralize pancreatic surgery care have reduced the postoperative mortality rate to less than 5%. However, the postoperative complication rate remains high, ranging from 40% to 50% (6–9).

The most common complication of pancreatoduodenectomy is postoperative pancreatic fistula (POPF), which has been shown to be one of the most intractable complications and can increase hospitalization costs and mortality (10, 11). Studies have shown that the occurrence of POPF is related to some important factors (12, 13), including the texture of the pancreas, blood supply to the tissues, the diameter of the main pancreatic duct (MPD), the quality of pancreaticojejunostomy (PJ), and the surgeon's experience; PJ is an independent risk factor for POPF (14).

It has been recognized that reconstruction after PD is technically challenging, and pancreaticojejunostomy methods and techniques are the main influential factors in pancreatic fistula. However, surgeons can improve the technical proficiency of pancreaticojejunostomy reconstruction by choosing a suitable anastomotic method and improving the quality of anastomosis (15). Therefore, a potential method of promoting surgeon proficiency in pancreatic anastomosis is to design a simplified and safe technique for this challenging reconstruction.

Biological healing is a novel concept of PJ that has been proposed by numerous surgeons in recent years (16–19). This novel theory emphasizes factors such as the blood supply of the tissues, the tension of the anastomotic stoma, healing of the pancreatic stump, and recovery of digestive function. “Wide, loose, and sparse” anastomosis has been recommended as a novel goal for PJ. Based on scholars Bassi and Miao's method (20, 21). With this novel theory of “biological healing,” we developed a novel and innovative anastomotic method: end-to-side one-layer continuous pancreaticojejunostomy. Twenty-five patients have been treated with this novel method since 2020. As such, we conducted this single-center retrospective study to compare the clinical values and outcomes of PD patients undergoing end-to-end invaginated pancreaticojejunostomy with those undergoing the novel end-to-side one-layer continuous pancreaticojejunostomy.

## Materials and methods

### Patients and data

In this single-center retrospective trial, 65 patients with pathologically confirmed lesions in the pancreatic head and surrounding areas who underwent PD by either end-to-end invaginated pancreaticojejunostomy (Group A) or end-to-side one-layer continuous pancreaticojejunostomy (Group B) from September 2020 to December 2021 at the Department of General Surgery, Xiangya Hospital, Central South University, were enrolled.

The inclusion criteria were as follows: (1) adult patients (age from 18 to 80 years); (2) planned for selective pancreaticoduodenectomy; (3) no distant metastasis (including pelvic cavity, peritoneum, liver, lung, brain, bone, etc.) determined by ultrasound or CT; (4) not receiving radiotherapy and chemotherapy before surgery; (5) no history of other malignant tumors or associated with other organ dysfunction.

The exclusion criteria were as follows: (1) MPD could not be identified intraoperatively; (2) change to other surgical procedures, such as total pancreatectomy or segmental resection; (3) external drainage was added or occlusion of the MPD occurred for any reason; (4) resection combined with other organs; (5) pancreaticoduodenectomy combined with vascular resection and laparoscopic resection patients.

Clinical data, including baseline demographic characteristics, operation duration, and complications (including pancreatic fistula, intraperitoneal infection, intraperitoneal bleeding, reoperation, postoperative hospital stay, and perioperative death) were collected (Tables 1, 2).

**Table 1:** The general information of two groups.

Variables	End-to-end sleeve anastomosis (*n* = 40)	End-to-side one-layer continuous anastomosis (*n* = 25)	*p*-value
Gender			0.601
Male	24	15	
Female	16	10	
Age (years)	52.3 ± 11.4	55.1 ± 10.9	0.947
Primary disease			1.000
Pancreatic head carcinoma	13	8	
Ampullary carcinoma	16	10	
Chronic pancreatitis	2	1	
Other	9	6	

**Table 2:** Postoperative complications in two groups.

Variables	End-to-end sleeve anastomosis (*n* = 40)	End-to-side one-layer continuous anastomosis (*n* = 25)	*p*-value
Pancreaticojejunostomy duration	32.6 ± 5.1 min	8.3 ± 2.2 min	<0.001
Pancreatic fistula	6 (15%)	3 (12%)	1.000
Grade A	3 (7.5%)	2 (8%)	
Grade B	2 (5%)	1 (4%)	
Grade C	1 (2.5%)	0 (0%)	
Intraperitoneal infection	1 (2.5%)	0 (0%)	1.000
Intraperitoneal bleeding	1 (2.5%)	0 (0%)	1.000
Reoperation	1 (2.5%)	0 (0%)	1.000
Postoperative hospital stay (days)	15.6 ± 6.1	14.8 ± 4.9	0.873
Perioperative death	1 (2.5%)	0 (0%)	1.000

All operations were performed by the same highly experienced and qualified surgeon (more than 35 years of clinical experience in pancreaticoduodenectomy) in the Department of General Surgery, Xiangya Hospital, Central South University. All work was reviewed and approved by the Ethics Committee of the Medical Council of Xiangya Hospital, Central South University. All patients or their legal representatives signed informed consent forms prior to surgery. According to Chinese law, this work was considered a quality-assured activity.

### Surgical procedure

**Figure 1:**
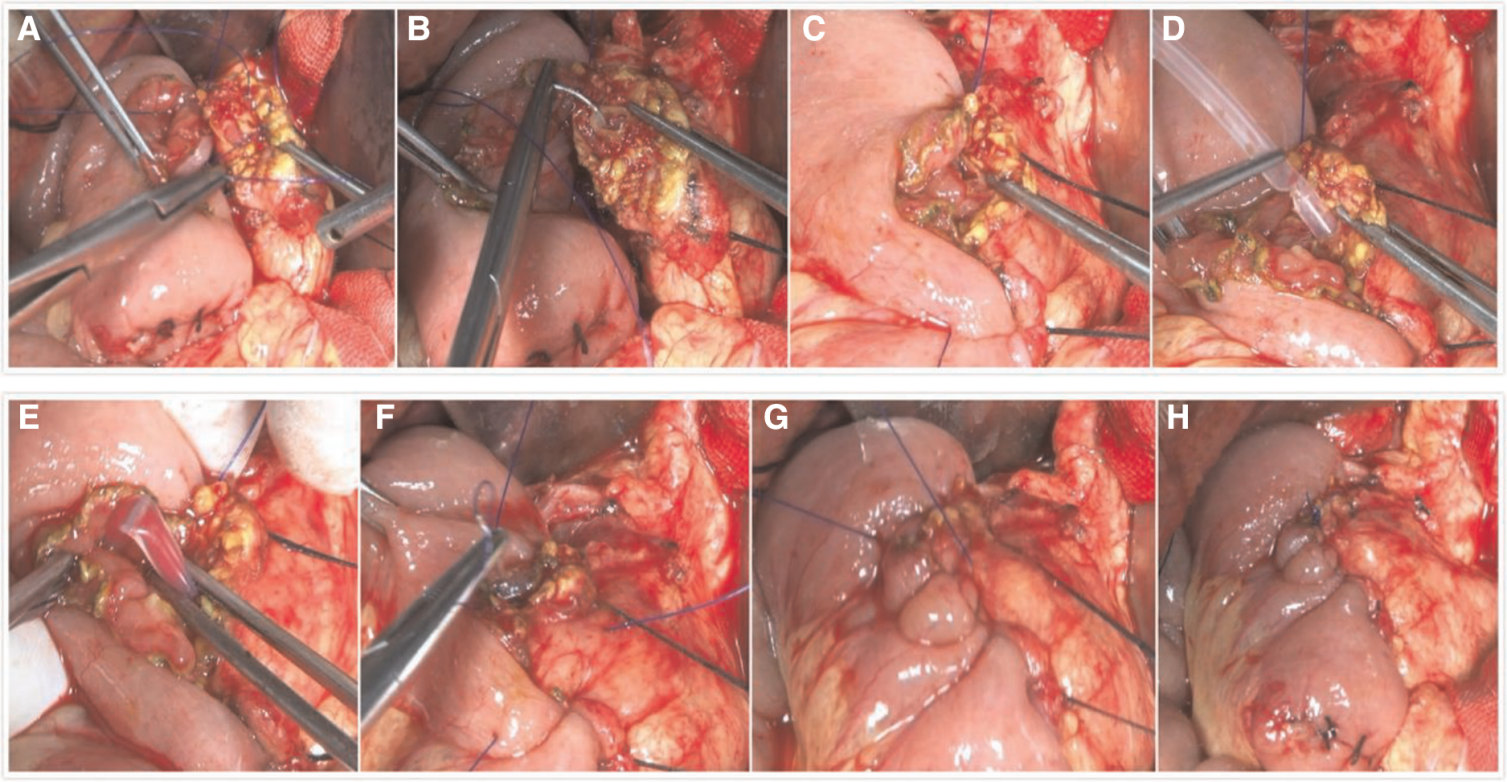
Intraoperative photographs of the novel end-to-side one-layer continuous pancreaticojejunostomy. (**A**) Perform all-layer continuous inverting suture between pancreatic margin and jejunal from the rear edge of the pancreas start with a 2-0 Prolene slip line. (**B**) Sew 2–3 stitches in the rear wall of the pancreatic duct and the posterior tissue together with the whole layer of the jejunum. (**C**) The suture of the rear wall is completed. (**D**) Build a support tube into the main pancreatic duct. (**E**) Put the support tube into the jejunum. (**F**) Turn to the front edge from the rear edge and sew the front edge of the pancreas and the other side of the jejunum with whole-layer suturing. (**G**) Take up the line. (**H**) Knot and complete pancreaticojejunostomy.

All patients underwent pancreaticoduodenal resection. In accordance with the principle of radical cancer cure, we performed an operation to remove the entire tumor; clear the lymph node; skeletonize the hepatoduodenal ligament, the portal vein, and the superior mesenteric artery; and remove retroperitoneal tissue. The end-to-end pancreaticojejunostomy sleeve anastomosis used conventional child anastomosis. The method for the novel end-to-side one-layer continuous pancreaticojejunostomy was as follows. The surgeon made an all-layer continuous inverting suture between the pancreatic margin and the jejunum from the rear edge of the pancreas. Starting with a 2-0 Prolene slip line, the spacing was approximately 8–10 mm, and the margin was greater than 10 mm. Then, a support tube was built into the main pancreatic duct. When the rear wall was sutured, we placed the support tube into the jejunum. If the main pancreatic duct is greater than 4 mm in diameter, 2–3 stitches were sewed in the rear wall of the pancreatic duct and the posterior tissue together with the entire layer of jejunum. The front edge was turned from the rear edge, and the front edge of the pancreas and the other side of the jejunum were sewed with whole-layer suturing. The line was followed and knotted with the first line, and then pancreaticojejunostomy was completed. The critical points of our anastomotic technique included proper tension in the suture: not pulling too tightly in order to avoid pancreatic laceration, covering the entire pancreatic stump with the jejunal wall, not leaving dead space in between, and ensuring good contraposition of the opening of the jejunal wall (Figure 1).

### Postoperative management

All patients received routine medicine administration to prevent infection, suppress gastric acid, inhibit pancreatic secretion, protect liver function, support nutrition, and receive treatment for complications. Prophylactic octreotide was pumped continuously to all patients for 72 h after surgery. The amylase level of the drainage fluid was measured on postoperative days 1, 3, and 5 per the routine protocol and thereafter according to the surgeon's need.

### Postoperative complications

Postoperative complications of PD mainly include pancreatic fistula, postoperative intraperitoneal hemorrhage, anastomotic bleeding, biliary fistula, intestinal fistula, gastric emptying dysfunction, intraperitoneal infection, and so on. The diagnosis of pancreatic fistula standard adopts the International Team of Pancreatic Fistula (International Study Group of Pancreatic Fistula, ISGPF) definition of pancreatic fistula from 2005 (22): 3 days or more after surgery, amylase of drainage fluid from the drainage tube of surgical placement (or of subsequent percutaneous placement) is three times higher than the normal serum amylase limit. Patients with pancreatic fistula were divided into levels A, B, and C according to the clinical effect (Supplementary Table S1).

### Statistical analysis

The characteristics of the patients were summarized with frequencies and percentages (for categorical variables) or mean values ± standard deviations. SPSS 27.0 software (IBM Corporation, New York, United States) was used for data analysis. The measurement data were tested using the *t*-test, and the categorical data were tested by *χ*^2^ test. *p *< 0.05 indicated a significant difference.

## Results

Sixty-five patients who underwent PD were included from September 2020 to December 2021: 40 patients underwent end-to-end invaginated pancreaticojejunostomy, and 25 underwent the novel end-to-side one-layer continuous pancreaticojejunostomy approach. There were no significant differences in age, sex, and primary disease of the patients between groups (*p* > 0.05). The baseline characteristics were also similar between groups (Table 1).

No significant difference was observed in the rates of pancreatic fistula, intraperitoneal infection, intraperitoneal bleeding, reoperation, postoperative hospital stay, and perioperative death. However, the pancreaticojejunostomy duration was significantly shorter (8.3 ± 2.2 vs. 32.6 ± 5.1 min) in the novel end-to-side one-layer continuous pancreaticojejunostomy group than in the end-to-end invaginated pancreaticojejunostomy group (*p* < 0.001). The incidence of pancreatic fistula in the novel pancreaticojejunostomy was 12%, including two cases of grade A pancreatic fistula, one case of grade B pancreatic fistula, and no cases of grade C pancreatic fistula. No deaths occurred during the perioperative period (Table 2).

## Discussion

POPF is one of the most common and severe postoperative complications, and it can lead to a prolonged postoperative recovery time, intraperitoneal infection, intraperitoneal bleeding, and other complications. The onset of POPF can increase the mean length of hospital stay and medical costs, resulting in poor quality of life or even death (23, 24), which has been a main clinical challenge for pancreatic surgeons. Therefore, the healing of pancreatic-enteric anastomosis becomes very important for the prevention of pancreatic fistula.

Anastomosis between digestive organs, although under unique influences, such as digestive juice, motility, and tension, has a basic wound healing process, which can be divided into three periods (25): the inflammatory phase, proliferative phase, and remodeling phase. The inflammation phase usually occurs 0–7 days after surgery and presents mainly as local aggregation and infiltration of inflammatory cells. The proliferative neovascular response also performed relatively actively in this period. This period is prone to be accompanied by anastomotic leakage due to necrosis, bleeding, loss, and incomplete repair of the anastomotic tissue. The proliferative phase is generally 7–14 days after surgery. In this phase, inflammatory cells engulf necrotic tissue with significantly reduced leakage, an obvious proliferation of granulation tissue, an increasing number of fibroblasts, and large amounts of collagen fibers produced to repair wounds. In the remodeling phase, which occurs between 3 weeks and approximately 2 months after surgery, there is a further increase and gradually ordered collagen fibers that firm wound healing.

Pancreatic anastomotic healing has its specialty. First, the pancreas is a solid organ with slight toughness and can easily be torn when sutured. Second, anastomosis between the jejunum and pancreas, i.e., healing between different tissues, is influenced by pancreatic juice, bile, intestinal juice, and other types of digestive juice, which can be accompanied by severe necrosis and inflammatory exudation, long organization time, and slow epithelial regeneration. Therefore, anastomosis between the jejunum and pancreas has a markedly longer healing time than intestinal anastomosis, especially when the pancreas is soft with a small duct, which is a known risk factor for POPF.

Most clinicians believe that the novel anastomosis technique for pancreaticojejunostomy decreases the incidence of POPF in PD (26, 27). Therefore, surgeons have been concerned about exploring novel pancreaticojejunostomy techniques.

Since March 2020, we have performed a novel pancreaticojejunostomy method—pancreas–intestinal end-to-side one-layer continuous anastomosis—based on research related to full mouth whole-layer interrupted anastomosis that was conducted by Bassi, Miao ,and other scholars (20, 21).

Twenty-five patients underwent this novel pancreaticojejunostomy approach and achieved good results. No significant differences were observed in pancreatic fistula, intraperitoneal infection, intraperitoneal bleeding, reoperation, postoperative hospital stay, and perioperative death between the novel end-to-side one-layer continuous pancreaticojejunostomy group and the end-to-end invaginated pancreaticojejunostomy group.

Relevant studies have reported that the prevalence of POPF ranges from 10% to 40%, and a fistula rate of approximately 30% is generally accepted (28, 29). In our study, the incidence of PF in the novel pancreaticojejunostomy group was 12%, including two cases of grade A POPF, one case of grade B POPF, and no cases of grade C POPF. All patients with pancreatic fistula recovered after conservative treatment. There were no perioperative deaths.

Traditional pancreaticojejunostomy often attempts to reduce the occurrence of pancreatic fistula by using secure or even more stitched layers that are mechanically connected to the anastomosis. However, this anastomosis inhibits the natural biological healing process of pancreaticojejunostomy, so the incidence of postoperative pancreatic fistula after pancreaticojejunostomy does not decrease along with the various changes in the anastomosis method. Our understanding is that the goal of pancreaticojejunostomy is to establish pancreaticojejunostomy continuity between the pancreas and the jejunum by inducing biological healing (30). Stitching itself provides only the necessary conditions for the spatial proximity of a biological connection and the subsequent healing of organizations. A consistent and good approach should meet the following conditions: (1) anastomotic tissue has good blood supply; (2) margin involution is good; (3) damage to tissue cutting is minimal; and (4) it is simple and easy to operate. Thus, pancreaticojejunostomy could provide good conditions for healing.

The novel end-to-side one-layer continuous pancreaticojejunostomy group had significantly shorter pancreaticojejunostomy duration than the end-to-end invaginated pancreaticojejunostomy group. The novel method can be done within 6–10 min by experienced surgeons. In summary, this novel end-to-side one-layer continuous pancreaticojejunostomy is simpler and less time-consuming than the traditional method.

In clinical practice, we recognize that the end-to-side one-layer continuous pancreaticojejunostomy technique is superior with respect to simplicity and reliability. (1) Using Prolene slip lines (nonbiodegradable sutures that can maintain permanent tensile strength after being implanted into the tissue, extend with the creeping of the organization, and do not split due to fatigue) to suture a single layer continuously with an exact degree of sparse guarantees anastomosis and a better blood supply. This is a superior approach to try to do a tight suture with the line. (2) An appropriate degree of take-up and knotting, rather than cutting pancreatic tissue, can make the anastomosis margin moderately closer and keep the jejunum mucosa and pancreas margin neatly fit in the space, providing a good anatomic and physiological environment for healing. (3) Single-layer continuous sutures, rather than interrupted sutures, prevent tissue fragmentation due to repeated knotting (especially those with a soft pancreatic texture). The more complex the suture is, the more likely it is to affect the blood supply of the anastomosis, prolonging the phase of anastomotic inflammation and fiber decomposition and consequently leading to pancreatic fistula. Single continuous sutures shorten the phase of inflammation and fiber decomposition to make pancreaticojejunostomy heal faster with less scarring and reduced incidence of pancreatic fistula. (4) Single continuous sutures make the tension between the suture and anastomosis organizations distribute uniformly and softly so that the overall anti-tensile strength of anastomosis is high. (5) The intraductal support tube drains pancreatic secretin into the jejunum instead of accumulating in the anastomosis.

Our study has some limitations. First, as mentioned above, our sample size was relatively small. Second, this was a single-center, retrospective study because this novel pancreaticojejunostomy has been modified and is performed in our hospital currently. Therefore, multicenter randomized trials are needed for further research.

## Conclusions

In conclusion, we found that the novel end-to-side one-layer continuous pancreaticojejunostomy did not increase the rate of postoperative complications after PD. However, the novel end-to-side one-layer continuous pancreaticojejunostomy showed advantages such as shorter pancreaticojejunostomy duration and a potentially reduced prevalence of pancreatic fistula. The findings need to be further validated with additional observational studies and animal experiments with large sample sizes. This novel method is feasible in both theory and practice. It is worthy of promotion and may bring significant clinical advantages to PD patients.

## Data Availability

The original contributions presented in the study are included in the article/Supplementary Material, further inquiries can be directed to the corresponding authors.
