# Supplementary material for: Novel end-to-side one-layer continuous pancreaticojejunostomy vs. end-to-end invaginated pancreaticojejunostomy in pancreatoduodenectomy: A single-center retrospective study
Source: Front Surg. 2023 Jan 6;9:980056. doi: 10.3389/fsurg.2022.980056 (PMC9852522; doi:10.3389/fsurg.2022.980056)
Supplement: Supplementary file 1 [file Table1.docx]

**Supplement Table 1 Pancreatic fistula classification**

| Gradient basis | Grade A | Grade B | Grade C |
| --- | --- | --- | --- |
| Clinical manifestations | Good | OK. | onset of symptoms/poor |
| Specific Treatment | No | Yes/ No | Yes. |
| US/CT | Negative | negative/positive | positive |
| Continuous drainage 3 weeks after surgery | No | Usually yes | Yes. |
| Reoperation | No | No | Yes. |
| Pancreatic Fistula-Related Death | No | No | Maybe yes |
| Signs of infection | No | Yes. | Yes. |
| Sepsis | No | No | Yes. |
| Readmission | No | Yes /no | Yes/no |
